# Supplementary material for: A thorough analysis of the contribution of experimental, derived and sequence-based predicted protein-protein interactions for functional annotation of proteins
Source: PLoS One. 2020 Nov 25;15(11):e0242723. doi: 10.1371/journal.pone.0242723 (PMC7688180; doi:10.1371/journal.pone.0242723)
Supplement: S1 File — (PDF) [file pone.0242723.s001.pdf]

A thorough analysis of the contribution of experimental, derived and sequence-based predicted protein-protein interactions for functional annotation of proteins

S1 File

Stavros Makrodimitris [1,2,†], Marcel J.T. Reinders [1,3], Roeland C.H.J. van Ham [1,2]

[1]Delft Bioinformatics Lab, Delft University of Technology, Delft, the Netherlands,
[2]Keygene N.V., Wageningen, the Netherlands and
[3]Leiden Computational Biology Center, Leiden University Medical Center, Leiden, the Netherlands.
[†]To whom correspondence should be addressed.

Statistics of PPI networks

The experimental PPI networks of the four species have very different degree distributions, as shown in Figure S1. Table S1 lists the number of total and unique protein-protein interactions (PPIs) that each data source includes for *Saccharomyces cerevisae*, *Escherichia coli*, *Arabidoisis thaliana* and *Solanum lycopersicum.*

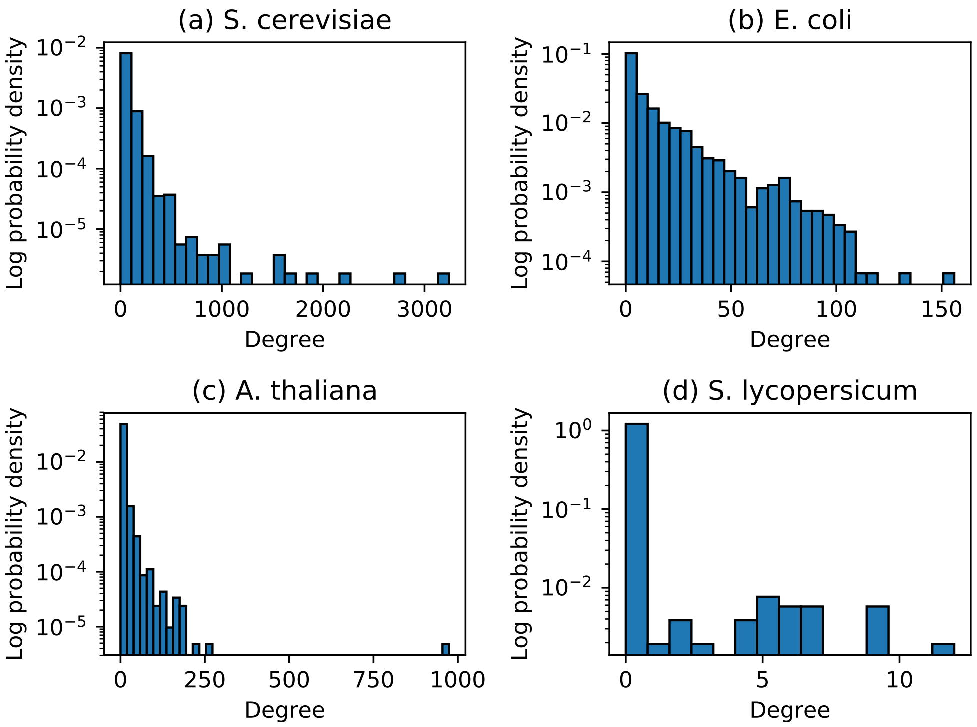

Figure S1: Histogram of node degrees in the experimental PPI networks of yeast (a), *E. coli* (b), arabidopsis (c) and tomato (d). On the x-axis node degree and on the y-axis the probability density in log scale.

Table S1: Number of interactions added by each data source (rows) in each species (columns). "Database" from STRING was not considered, as it includes protein associations based on known GO annotations

Data source	*S. cerevisiae*		*E. coli*		*A. thaliana*		*S. lycopersicum*	
	Total	Unique	Total	Unique	Total	Unique	Total	Unique
Physical (BIOGRID)	97,503	51,473	9,734	5,295	21,720	10,826	57	11
Experiments (STRING)	87,378	24,485	11,269	3,944	10,176	746	0	0
Neighborhood (STRING)	0	0	3,644	638	0	0	0	0
Neighborhood transferred (STRING)	55,410	30,241	49,167	27,128	165,627	106,821	117	57
Co-Occurence (STRING)	2,217	498	43,114	29,663	21,283	12,968	1,014	187
Database transferred (STRING)	0	0	0	0	0	0	0	0
Experiments transferred (STRING)	121,858	57,464	13,356	6,175	351,488	236,893	2,878	1,247
Fusion (STRING)	1,784	1,007	1,568	224	2,010	671	2	0
Homology (STRING)	3,700	561	1,083	414	31,983	16,734	1,858	212
Co-Expression (STRING)	71,525	24,635	0	0	285,075	217,555	0	0
Co-Expression transferred (STRING)	242,228	100,849	47,897	21,878	390,727	193,802	3,029	622
Text mining (STRING)	206,721	122,099	7,609	4,798	222,844	171,286	904	595
Text mining transferred (STRING)	211,409	95,945	56,745	30,854	497,495	313,994	3,712	1,412

Integration of STRING scores

For completeness, we describe the algorithm for integrating STRING scores from D different sources [1]. We use s_i to denote the score (probability) of a particular interaction based on data type i. This score is computed by taking into account the prior probability of two proteins interacting (p), for which the value $p = 0.041$ is used. When combining multiple data types, this prior score should be only be incorporated once, so the first step is to remove it from all data types (Eq 1):

$$s_{i,noprior} = \frac{s_i - p}{1 - p} \tag{1}$$

Scores from different sources are combined using Eq 2:

$$s_{combined,noprior} = 1 - \prod_{i=1}^{D}(1 - s_{i,noprior}) \tag{2}$$

Finally, the prior probability is incorporated back into the score by Eq 3, which is the same as Eq 1, but solving for a different variable.

$$s_{combined} = s_{combined,noprior} + p(1 - s_{combined,noprior}) \tag{3}$$

Evaluation measures and results

Definitions

We evaluated using the protein-centric F1 score and Semantic Distance (SD) [2]. For completeness, we provide definitions of these measures using notation similar to [3]: T_i is the set of GO terms annotated for protein i and $P_i(\tau)$ is the set of terms predicted for protein i with posterior probability larger than τ. C denotes the number of GO terms and n the number of proteins

F1 score is calculated from precision (equation 4) and recall (equation 5) from equation 6. Semantic Distance is calculated based on Remaining Uncertainty (ru, equation 7) and Misinformation (mi, equation 8) using equation 9 [2]. ru quantifies the information content of the false negatives and misinformation that of the false positives.

$$pr(\tau) = \frac{1}{n}\sum_{i=1}^{n}\frac{\sum_{t=1}^{C} I(t \in P_i(\tau) \wedge t \in T_i)}{\sum_{t=1}^{C} I(t \in P_i(\tau))} \tag{4}$$

$$rc(\tau) = \frac{1}{n}\sum_{i=1}^{n}\frac{\sum_{t=1}^{C} I(t \in P_i(\tau) \wedge t \in T_i)}{\sum_{t=1}^{C} I(t \in T_i)} \tag{5}$$

$$F1(\tau) = 2\frac{pr(\tau) \cdot rc(\tau)}{pr(\tau) + rc(\tau)} \tag{6}$$

$$ru(\tau) = \frac{1}{n}\sum_{i=1}^{n} IC(t) \cdot I(t \in T_i \wedge t \notin P_i(\tau)) \tag{7}$$

$$mi(\tau) = \frac{1}{n}\sum_{i=1}^{n} IC(t) \cdot I(t \notin T_i \wedge t \in P_i(\tau)) \tag{8}$$

$$SD(\tau) = \sqrt{ru(\tau)^2 + mi(\tau)^2} \tag{9}$$

All these measures are calculated using hard (0 or 1) predictions and not posterior probabilities. Similar to the CAFA challenges [4], we report the metric value of each method for the optimal operating point τ (selected on the test data), considering all operating points from 0 to 1 with a step 0.02 (equations 10 and 11). Conversely to F1 score, lower Semantic Distance corresponds to better performance.

$$F_{max} = \max_{\tau \in [0,1]} F1(\tau) \tag{10}$$

$$S_{min} = \min_{\tau \in [0,1]} SD(\tau) \tag{11}$$

Results

The performance of all tested methods is listed in Tables S2 and S3.

Table S2: $F_{\max}$ of different function prediction methods (rows) in 4 species (columns) estimated using 5-fold cross-validation. The mean ±the standard deviation is shown.

	S. cerevisiae	*E. coli*	*A. thaliana*	*S. lycopersicum*
Naive	0.31±0.004	0.29±0.009	0.28±0.005	0.23±0.019
BLAST	0.35±0.004	0.43±0.021	0.42±0.007	0.34±0.025
EXP, GBA	0.42±0.007	0.25±0.011	0.19±0.007	0.08±0.007
EXP, node2vec	0.50±0.012	0.28±0.011	0.23±0.008	0.08±0.042
EXP, GBA + BLAST	0.50±0.007	0.45±0.019	0.42±0.006	0.33±0.025
EXP + STRING, GBA	0.49±0.005	0.46±0.008	0.48±0.004	0.61±0.045
EXP + STRING, node2vec	0.59±0.009	0.50±0.012	0.50±0.005	0.61±0.042
EXP + STRING, GBA + BLAST	0.54±0.006	0.58±0.021	0.54±0.008	0.60±0.047
EXP + SEQ, GBA	0.33±0.005	0.32±0.008	0.29±0.004	0.33±0.016

Table S3: S_{min} of different function prediction methods (rows) in 4 species (columns) estimated using 5-fold cross-validation. The mean ±the standard deviation is shown.

	S. cerevisiae	*E. coli*	*A. thaliana*	*S. lycopersicum*
Naive	34.67±0.75	21.41±0.20	30.44±0.57	17.67±0.77
BLAST	35.24±0.97	16.85±0.85	27.66±0.31	19.31±0.87
EXP, GBA	31.17±0.55	21.58±0.44	30.55±0.61	19.39±0.91
EXP, node2vec	30.05±0.55	20.37±0.41	28.69±0.59	19.37±0.89
EXP, GBA + BLAST	29.28±0.83	15.57±0.79	27.75±0.55	18.25±0.92
EXP + STRING, GBA	27.42±0.57	17.07±0.22	24.12±0.50	9.08±0.89
EXP + STRING, node2vec	25.65±0.48	17.23±0.34	25.10±0.15	9.20±0.77
EXP + STRING, GBA + BLAST	28.48±0.97	12.94±0.60	23.00±0.56	10.21±1.06
EXP + SEQ, GBA	34.50±0.74	21.02±0.14	30.48±0.57	17.06±0.71

Table S4: F_{max}, S_{min} and coverage of the *node2vec* method on the EXP PPI network in four species when excluding and including proteins without any functional annotations during the feature learning step. Where one of the two approaches is significantly (FDR $<$ 5%) better, it is shown in bold.

Species	F_{max}		S_{min}		Coverage	
	Annotated proteins only	All proteins	Annotated proteins only	All proteins	Annotated proteins only	All proteins
S. cerevisiae	0.50 ± 0.012	0.50 ± 0.010	30.05 ± 0.54	30.13 ± 0.32	0.99 ± 0.002	0.99 ± 0.001
E. coli	0.28 ± 0.011	0.29 ± 0.008	20.37 ± 0.41	20.47 ± 0.31	0.77 ± 0.016	**0.79 ± 0.017**
A. thaliana	0.23 ± 0.008	0.23 ± 0.009	28.69 ± 0.49	28.94 ± 0.49	0.57 ± 0.010	**0.58 ± 0.011**
S. lycopersicum	0.08 ± 0.007	0.08 ± 0.007	19.37 ± 0.89	19.36 ± 0.89	0.03 ± 0.017	0.03 ± 0.017

Comparison of GBA and *node2vec* per protein

Table S5: P-values before and after multiple testing correction to assess the significance in the median per-protein performances of GBA and *node2vec* at different levels of node degree. The bin numbers correspond to the bins shown in Figure 3 in the main document, with bin 1 denoting the smallest number of neighbors. For species where fewer bins were used, a dash signifies unused bins.

Bin number	*S. cerevisiae*		*E. coli*		*A. thaliana*	
	raw	corrected	raw	corrected	raw	corrected
1	0.028	0.036	7.5×10^{-11}	1.3×10^{-10}	$< 10^{-20}$	$< 10^{-20}$
2	1.6×10^{-5}	2.9×10^{-5}	1.8×10^{-11}	9.0×10^{-11}	$< 10^{-20}$	$< 10^{-20}$
3	0.845	0.845	7.6×10^{-11}	1.3×10^{-10}	$< 10^{-20}$	$< 10^{-20}$
4	0.004	0.006	0.031	0.031	4.4×10^{-16}	5.5×10^{-16}
5	7.4×10^{-6}	1.7×10^{-5}	1.9×10^{-5}	2.4×10^{-5}	0.001	0.001
6	2.5×10^{-21}	2.2×10^{-20}	-	-	-	-
7	5.5×10^{-12}	2.5×10^{-11}	-	-	-	-
8	5.5×10^{-6}	1.6×10^{-5}	-	-	-	-
9	0.109	0.123	-	-	-	-

node2vec parameters and tuning

The *node2vec* algorithm has several parameters that can have great influence on the final node embeddings [5] and therefore the function prediction performance. For each species, we tuned these parameters for the experimental network and for the best combination of experimental and STRING edges separately. To do so, we employed a grid search. Below is a list of the parameters we tuned and the ranges of parameter values we considered:

- The number of random walks per node ([5, 10, 15, 20, 30]),
- The parameter p [5] ([0.1, 0.5, 1.0, 1.5, 3.0]),
- The parameter q [5] ([0.1, 0.5, 1.0, 1.5, 3.0]),
- The length of each random walk ([20, 40, 60, 80, 100, 160]), and
- The dimensionality of the embedding vector ([32, 64, 96, 128, 150, 200, 500]).

For each of the combinations above, we tried the kNN and ridge classifiers. The kNN also has one tunable parameter, the number of neighbors k, for which we considered the values [1, 2, 5, 7, 9, 11, 15, 21, 31, 51]. The parameter λ of ridge, which controls the amount of L2 regularization, was tuned among the values [0.0001, 0.001, 0.01, 0.1, 0.5, 1.0, 2.0, 3.0, 5.0, 10.0].

This amounts to 105,000 classification models per network per species. Each model was evaluated in each of the five cross-validation folds, by further subdividing the training set of each fold into a training (80% of original training set) and a validation set (20% of originial training set). The combination of *node2vec* hyperparameters, classifier and classifier parameter that maximized the validation F_{max} in each fold was subsequently trained on the entire training set (of the corresponding fold) and the resulting trained model was tested on the previously unseen test set.

Removing edges from the yeast EXP PPI network

We started from the full *EXP* PPI network of yeast and step-wise removed 10%, 20%, 30%, . . . , 90% and 99% of its edges. The edges were removed at random, using two different strategies: In the first ("uniform"), the probability of removing an edge was uniform over all present edges. In the second ("degree"), it was inversely proportional to the smaller degree of the two nodes that the edge connects, so that nodes with the fewest edges are most likely to lose them. This procedure gave us 11 downsampled networks per sampling strategy on which we ran the GBA classifier, using the same cross-validation loop as before. We repeated this 5 times with different random seeds to obtain variability estimates. The performance is plotted against the amount of missing edges in Figure S2.

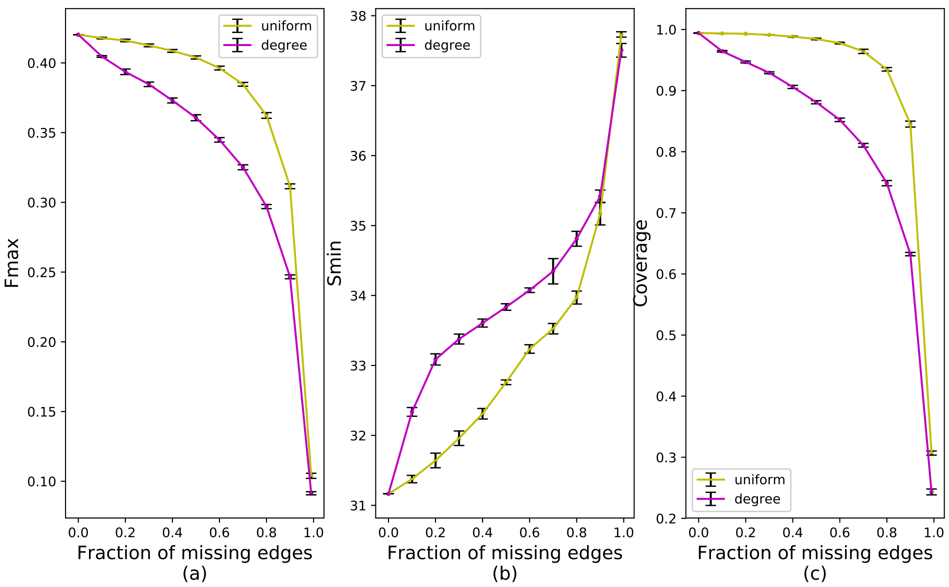

Figure S2: F_{max} (a), S_{min} (b) and coverage (c) of the GBA method on the yeast experimental PPI network (y-axis) as a function of the fraction of missing edges (x-axis). Edges where removed at random either uniformly (yellow) or inversely proportional to node degree, so that least connected nodes are more likely to lose their edges (purple). Error bars show standard deviation of 5 rounds of random sampling.

Using all STRING edges in a weighted graph

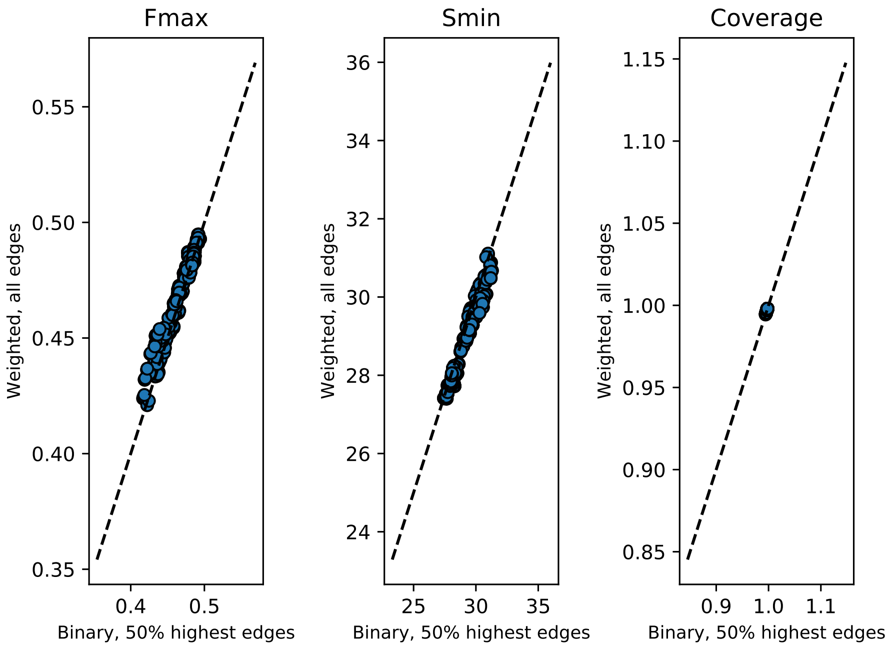

Figure S3: F_{max} (left), S_{min} (middle) and coverage (right) of the binary STRING networks with the 50% top edges (x-axis) against the weighted ones with all edges (y-axis) for *S. cerevisiae.* Each dot corresponds to one of the 511 combinations of the 9 STRING data sources added to the binary experimental network. The black dashed line shows the $y = x$ line to ease comparison.

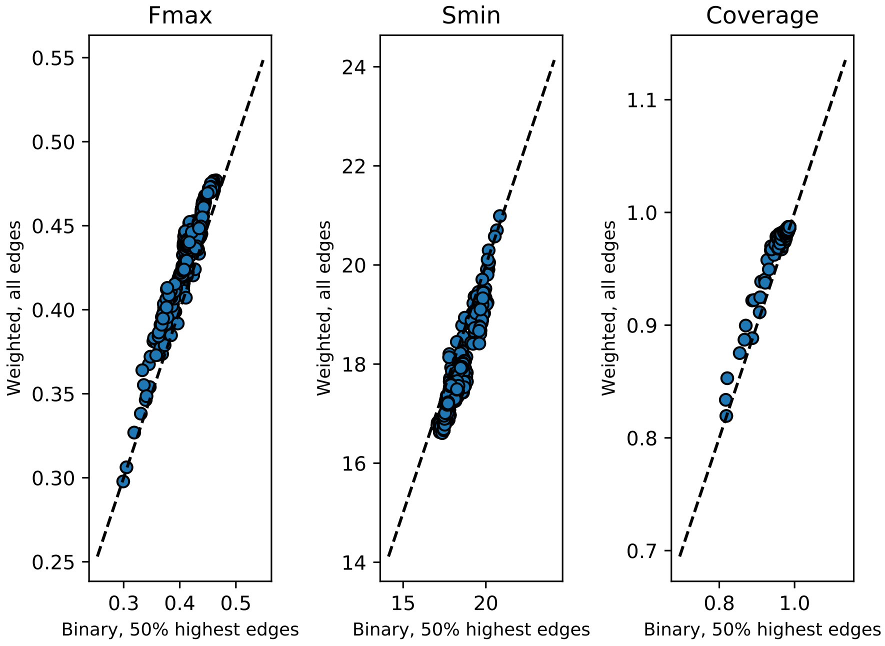

Figure S4: F_{max} (left), S_{min} (middle) and coverage (right) of the binary STRING networks with the 50% top edges (x-axis) against the weighted ones with all edges (y-axis) for *E. coli.* Each dot corresponds to one of the 511 combinations of the 9 STRING data sources added to the binary experimental network. The black dashed line shows the $y = x$ line to ease comparison.

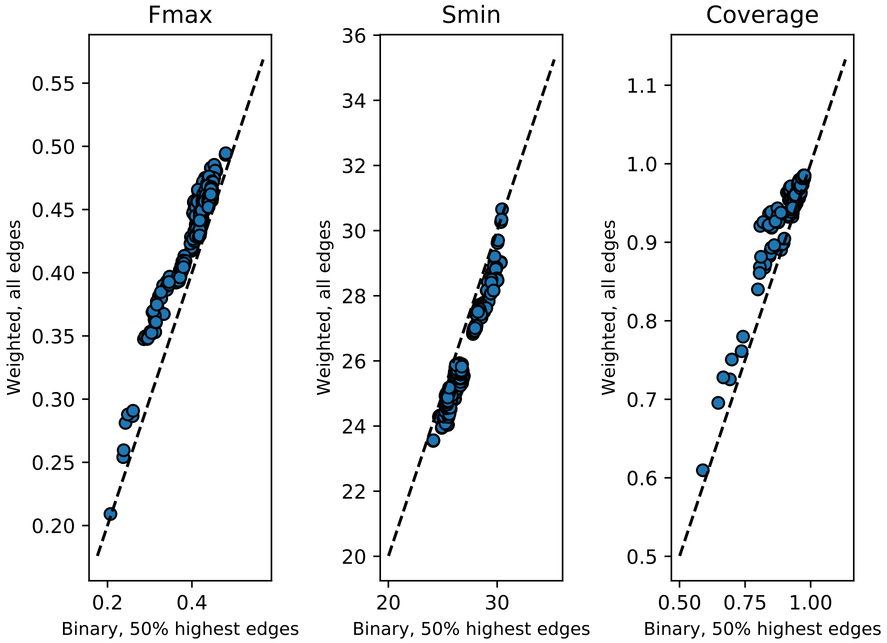

Figure S5: F_{max} (left), S_{min} (middle) and coverage (right) of the binary STRING networks with the 50% top edges (x-axis) against the weighted ones with all edges (y-axis) for *A. thaliana*. Each dot corresponds to one of the 511 combinations of the 9 STRING data sources added to the binary experimental network. The black dashed line shows the $y = x$ line to ease comparison.

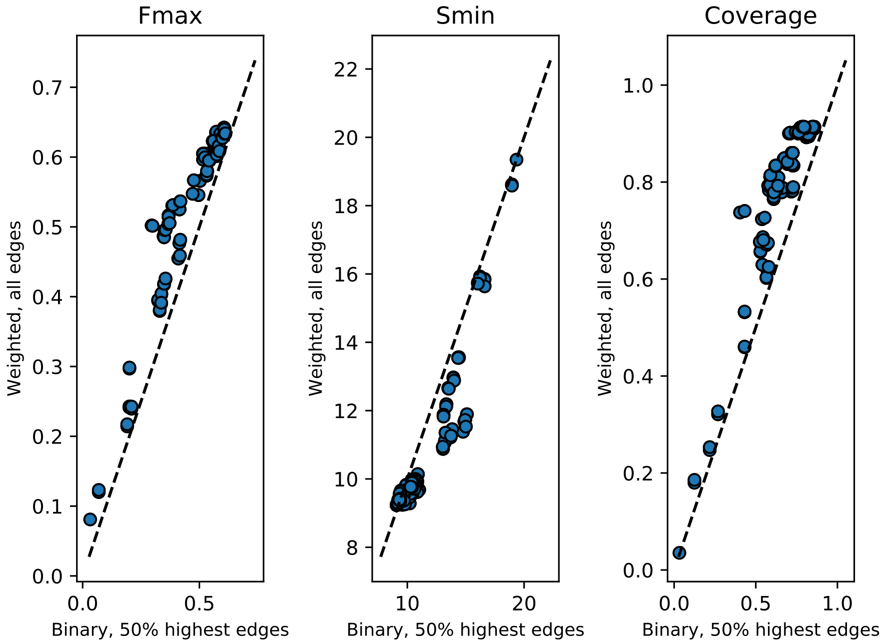

Figure S6: F_{max} (left), S_{min} (middle) and coverage (right) of the binary STRING networks with the 50% top edges (x-axis) against the weighted ones with all edges (y-axis) for *S. lycopersicum*. Each dot corresponds to one of the 255 combinations of the 8 STRING data sources added to the binary experimental network. The black dashed line shows the $y = x$ line to ease comparison.

Table S6: F_{max}, S_{min} and coverage of the *GBA* method on the *EXP+STRING* PPI network in four species when using a binary network with the top 50% STRING edges or a weighted one with all STRING edges. The performance of the best combination for each metric and each species is shown. Statistically significant differences (paired t-test, FDR < 0.05) are shown in bold.

Species	F_{max}		S_{min}		Coverage	
	Binary	Weighted	Binary	Weighted	Binary	Weighted
S. cerevisiae	0.49 ± 0.005	0.49 ± 0.005	27.43 ± 0.57	27.40 ± 0.61	0.99 ± 0.001	0.99 ± 0.001
E. coli	0.46 ± 0.008	$\mathbf{0.48 \pm 0.009}$	17.07 ± 0.22	16.61 ± 0.35	0.99 ± 0.004	0.99 ± 0.003
A. thaliana	0.48 ± 0.004	$\mathbf{0.49 \pm 0.005}$	24.12 ± 0.50	$\mathbf{23.54 \pm 0.50}$	0.98 ± 0.003	$\mathbf{0.99 \pm 0.001}$
S. lycopersicum	0.61 ± 0.045	0.64 ± 0.043	9.08 ± 0.89	9.23 ± 1.00	0.86 ± 0.020	$\mathbf{0.91 \pm 0.018}$

STRING performance per data source

For every species, we tested all possible combinations of *STRING* data sources and found that certain combinations perform significantly better than others, as certain individual data sources were more informative (Fig S7-S9.). To quantify the contribution of each data source we devised a statistical test described below.

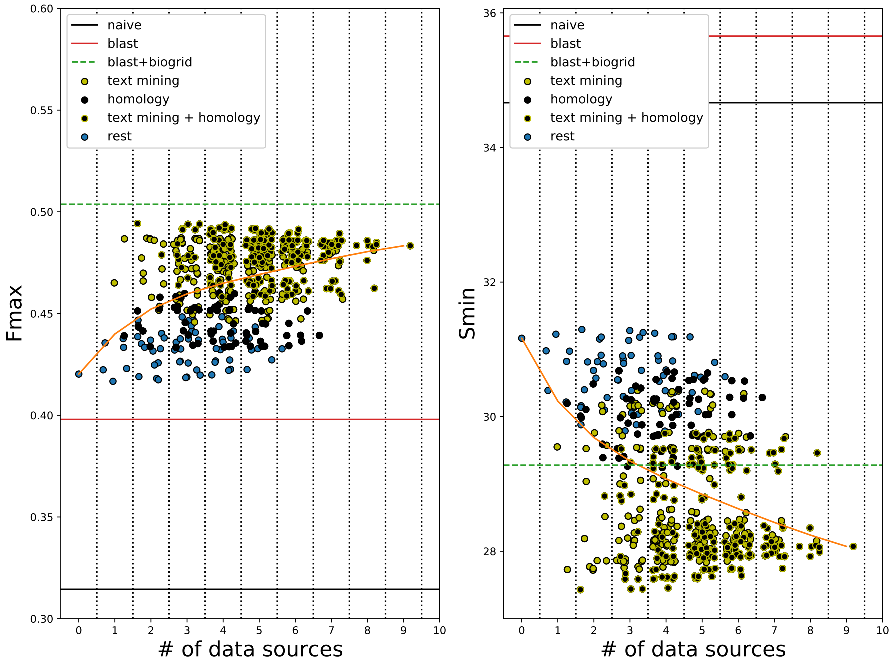

Figure S7: F_{max} (left) and S_{min} (right) in *S. cerevisiae* (y-axis) as a function of the number of *STRING* data sources included (x-axis). Each dot corresponds to one combination of data sources added to the experimental network. Combinations that include "text mining" and/or "text mining transferred" are shown in yellow, combinations that include "homology" in black and combinations that include both in black with yellow border. The rest of the combinations are shown in blue. To ease visibility, we added a random number in the range [-0.5, 0.5] to each combination of the same number of sources. Zero data sources corresponds to the *EXP* network and the orange line shows the average performance for a specific number of data sources. Horizontal lines denote the performance of the *naive* (black), *BLAST* (red) and the combination of *BLAST* with the *EXP* PPI network (dashed green).

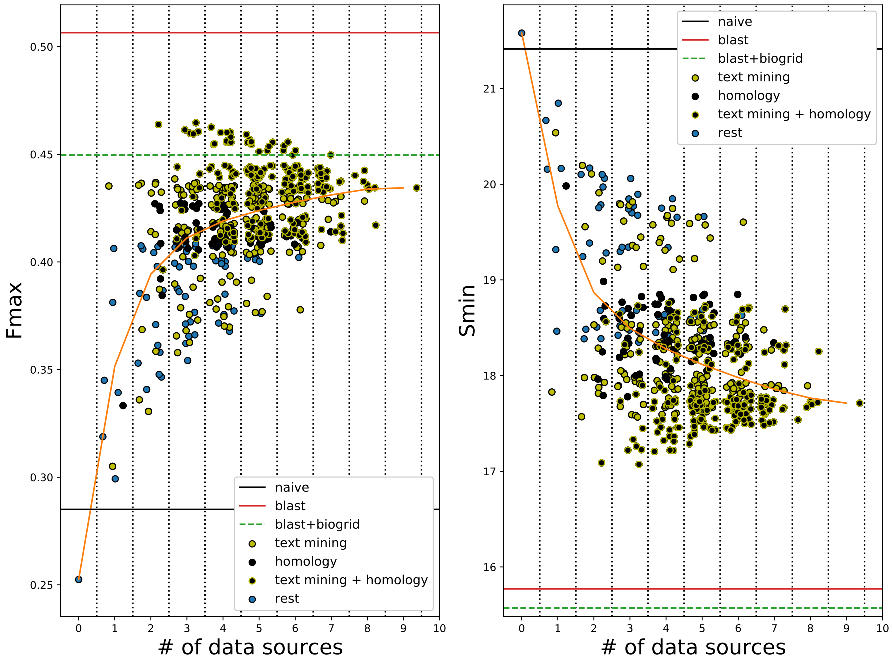

Figure S8: F_{max} (left) and S_{min} (right) in *E. coli* (y-axis) as a function of the number of *STRING* data sources included (x-axis). Each dot corresponds to one combination of data sources added to the experimental network. Combinations that include "text mining" and/or "text mining transferred" are shown in yellow, combinations that include "homology" in black and combinations that include both in black with yellow border. The rest of the combinations are shown in blue. To ease visibility, we added a random number in the range [-0.5, 0.5] to each combination of the same number of sources. Zero data sources corresponds to the *EXP* network and the orange line shows the average performance for a specific number of data sources. Horizontal lines denote the performance of the *naive* (black), *BLAST* (red) and the combination of *BLAST* with the *EXP* PPI network (dashed green).

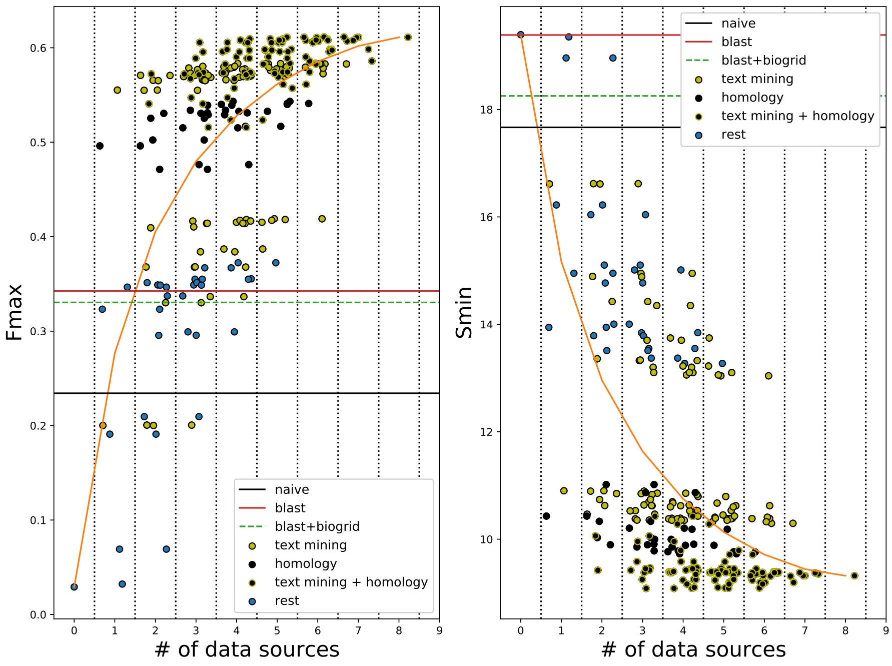

Figure S9: F_{max} (left) and S_{min} (right) in *S. lycopersicum* (y-axis) as a function of the number of *STRING* data sources included (x-axis). Each dot corresponds to one combination of data sources added to the experimental network. Combinations that include "text mining" and/or "text mining transferred" are shown in yellow, combinations that include "homology" in black and combinations that include both in black with yellow border. The rest of the combinations are shown in blue. To ease visibility, we added a random number in the range [-0.5, 0.5] to each combination of the same number of sources. Zero data sources corresponds to the *EXP* network and the orange line shows the average performance for a specific number of data sources. Horizontal lines denote the performance of the *naive* (black), *BLAST* (red) and the combination of *BLAST* with the *EXP* PPI network (dashed green).

For a species for which *STRING* contains protein associations from N different data sources, there are $2^N - 1$ ways to combine them (in combinations of different lengths). Each data source i is present in 2^{N-1} of these combinations (denoted as P_i) and absent in the remaining ones (denoted as A_i). We define an "irreplaceability" score ($IR(i)$) for i as the increase in the optimal performance of P_i with respect to A_i. For $\mathrm{F_{max}}$, this is formally defined in equation 12

$$IR_F(i) = \max_{c \in P_i} F_{max}(c) - \max_{c \in A_i} F_{max}(c) \tag{12}$$

An $IR_F(i)$ equal to 0, means that the combination that achieves the maximum performance does not include data source i, which implies that it made redundant by other sources. A positive value for $IR_F(i)$ means that i is necessary for maximizing *STRING* performance and a negative value that i is creating more errors than correct predictions. Since lower $\mathrm{S_{min}}$ means better performance, the definition of IR for $\mathrm{S_{min}}$ is slightly modified, so that a positive IR still denotes that a data source is useful (equation 13).

$$IR_S(i) = \min_{c \in A_i} S_{min}(c) - \min_{c \in P_i} S_{min}(c) \tag{13}$$

We compared the observed IR_F and IR_S values to what would be expected by chance to obtain a measure of statistical significance. To do so, we did a permutation test for which we randomly mixed the elements of P_i and A_i and calculated the IR_F and IR_S values again. We repeated this 10,000 times for each data source to obtain a null distribution of the two statistics and counted the fraction of times the permuted statistic was larger than the observed one. Note that this is a one-sided test, meaning that we only tested whether a data source was significantly irreplaceable, but not whether it was significantly leading to worse performance. We performed 90 tests in total, so we also applied multiple testing correction using the FDR method. The observed test statistics and the corresponding p-values are listed in Tables S7-S8 for yeast, S9-S10 for E. coli, S11-S12 for arabidopsis and S13-S14 for tomato.

Table S7: IR_F scores of STRING data sources, corresponding raw p-values and p-values corrected for multiple tests for *S. cerevisiae*

Data source	IR_F	p-value	corrected p-value
coexpression	-0.0026	0.9989	1.0
neighborhood transferred	-0.0024	0.9933	1.0
coexpression transferred	-0.0081	1.0	1.0
experiments transferred	-0.0078	1.0	1.0
**textmining**	0.0154	$< 10^{-4}$	< 0.007
cooccurence	-0.0004	0.8738900	1.0
fusion	-0.0002	0.75490	1.0
textmining transferred	-0.0022	0.9715	1.0
**homology**	0.0007	$< 10^{-4}$	< 0.007

Table S8: IR_S scores of STRING data sources, corresponding raw p-values and p-values corrected for multiple tests for *S. cerevisiae*

Data source	IR_S	p-value	corrected p-value
coexpression	-0.1572	0.9832	1.0
neighborhood transferred	-0.1571	0.9707	1.0
coexpression transferred	-0.4923	1.0	1.0
experiments transferred	-0.4663	1.0	1.0
**textmining**	1.3206	$< 10^{-4}$	< 0.007
cooccurence	-0.0105	0.7568	1.0
fusion	-0.0111	0.8798	1.0
textmining transferred	-0.1867	1.0	1.0
**homology**	0.2914	$< 10^{-4}$	< 0.007

Table S9: IR_F scores of STRING data sources, corresponding raw p-values and p-values corrected for multiple tests for *E. coli*

Data source	IR_F	p-value	corrected p-value
neighborhood	-0.0042	0.9695	1.0
neighborhood transferred	-0.0198	1.0	1.0
coexpression transferred	-0.0046	0.9962	1.0
experiments transferred	-0.0021	0.8759	1.0
textmining	-0.0045	0.9919	1.0
cooccurence	-0.0232	1.0	1.0
fusion	0.0008	0.4949	1.0
**textmining transferred**	0.0303	$< 10^{-4}$	< 0.007
**homology**	0.0261	$< 10^{-4}$	< 0.007

Table S10: IR_S scores of STRING data sources, corresponding raw p-values and p-values corrected for multiple tests for *E. coli*

Data source	IR_S	p-value	corrected p-value
neighborhood	-0.1456	0.9418	1.0
neighborhood transferred	-0.5245	1.0	1.0
coexpression transferred	-0.2496	0.9996	1.0
experiments transferred	-0.1348	0.8769	1.0
textmining	-0.1483	0.9839	1.0
cooccurence	-0.3763	1.0	1.0
fusion	0.016	0.4938	1.0
**textmining transferred**	0.6704	$< 10^{-4}$	< 0.007
**homology**	0.4951	$< 10^{-4}$	< 0.007

Table S11: IR_F scores of STRING data sources, corresponding raw p-values and p-values corrected for multiple tests for *A. thaliana*

Data source	IR_F	p-value	corrected p-value
coexpression	-0.0297	1.0	1.0
neighborhood transferred	-0.0225	0.96600	1.0
coexpression transferred	-0.0324	1.0	1.0
experiments transferred	-0.0275	1.0	1.0
**textmining**	0.0258	0.00010	0.0052
cooccurence	0.0001	0.50100	1.0
fusion	0.0002	0.25070	1.0
textmining transferred	-0.0252	0.9982	1.0
**homology**	0.0534	$< 10^{-4}$	< 0.007

Table S12: IR_S scores of STRING data sources, corresponding raw p-values and p-values corrected for multiple tests for *A. thaliana*

Data source	IR_S	p-value	corrected p-value
coexpression	-0.9484	1.0	1.0
neighborhood transferred	-0.7883	0.9984	1.0
coexpression transferred	-1.2361	1.0	1.0
experiments transferred	-1.1204	1.0	1.0
textmining	0.5247	0.0659	1.0
cooccurence	-0.0069	0.8801	1.0
fusion	-0.0066	0.7477	1.0
textmining transferred	-1.0243	1.0	1.0
**homology**	1.9112	$< 10^{-4}$	< 0.007

Table S13: IR_F scores of STRING data sources, corresponding raw p-values and p-values corrected for multiple tests for tomato

Data source	IR_F	p-value	corrected p-value
neighborhood transferred	-0.0002	0.98540	1.0
coexpression transferred	0.0019	0.00380	0.1938
**experiments transferred**	0.0031	$< 10^{-4}$	< 0.007
**textmining**	0.0128	$< 10^{-4}$	< 0.007
cooccurence	0.0002	0.24900	1.0
fusion	0.0000	0.75380	1.0
**textmining transferred**	0.0254	$< 10^{-4}$	< 0.007
**homology**	0.0281	$< 10^{-4}$	< 0.007

Table S14: IR_S scores of STRING data sources, corresponding raw p-values and p-values corrected for multiple tests for tomato

Data source	IR_S	p-value	corrected p-value
neighborhood transferred	-0.0029	0.93930	1.0
coexpression transferred	-0.0812	1.0	1.0
experiments transferred	-0.2280	1.0	1.0
**textmining**	0.1536	$< 10^{-4}$	< 0.007
cooccurence	-0.0085	0.9875	1.0
fusion	0.0000	0.75470	1.0
**textmining transferred**	0.2691	$< 10^{-4}$	< 0.007
**homology**	1.2114	$< 10^{-4}$	< 0.007

Removing the most informative data sources

Table S15: Changes in F_{max} of the combined *EXP+STRING* network, when removing text mining and/or homology edges

Data sources	*S. cerevisiae*	*E. coli*	*A. thaliana*	*S. lycopersicum*
All	0.49±0.005	0.46±0.008	0.48±0.004	0.61±0.045
No text mining/text mining transferred	0.46±0.006	0.43±0.013	0.45±0.007	0.54±0.053
No homology	0.49±0.004	0.44±0.011	0.43±0.006	0.58±0.044
No text mining/text mining transferred/homology	0.45±0.006	0.41±0.010	0.35±0.004	0.37±0.042

Table S16: Changes in S_{min} of the combined *EXP+STRING* network, when removing text mining and/or homology edges

Data sources	*S. cerevisiae*	*E. coli*	*A. thaliana*	*S. lycopersicum*
All	27.42±0.57	17.07±0.22	24.12±0.50	9.08±0.89
No text mining/text mining transferred	29.24±0.64	17.78±0.43	24.65±0.59	9.72±1.16
No homology	27.72±0.57	17.57±0.26	26.03±0.51	10.30±0.84
No text mining/text mining transferred/homology	29.79±0.63	18.35±0.22	28.47±0.47	13.27±0.65

PIPR training and results

Table S17: Effect of hyperparameters in training of PIPR for predicting yeast PPIs from the BIOGRID database.

**Optimizer**	**Learning Rate**	**Validation loss**	**Validation accuracy**
ADAM	0.001	0.507	0.771
ADAM	0.0001	0.540	0.754
SGD + learning rate scheduler	0.01	0.525	0.751
SGD + learning rate scheduler	0.001	0.547	0.737

The originally trained PIPR model in yeast generalized poorly in Arabidopsis (accuracy of 51% on a balanced dataset). Therefore, we chose to train PIPR on Arabidopsis by using the original trained model as initial conditions. We trained using Stochastic Gradient Descent with learning rate 0.001 and early stopping based on the validation loss with patience of 40 epochs. As validation set, we randomly selected 10% of the data and as loss function the binary cross-entropy. We did not use RMSprop optimizer, which was used by the authors, as it produced unstable results.

Table S18: Comparison of function prediction performance in arabidopsis and tomato of edges predicted with PIPR trained in either yeast or arabidopsis

Train species	Test species	F_{max}	S_{min}
S. cerevisiae	*A.thaliana*	0.29 ± 0.004	30.48 ± 0.57
A. thaliana	*A.thaliana*	0.29 ± 0.006	30.16 ± 0.29
S. cerevisiae	*S. lycopersicum*	0.33 ± 0.016	17.05 ± 0.70
A. thaliana	*S.lycopersicum*	0.35 ± 0.017	16.24 ± 0.66

References

[1] Christian von Mering et al. “STRING: Known and predicted protein-protein associations, integrated and transferred across organisms”. In: *Nucleic Acids Research* (2005). ISSN: 03051048. DOI: 10.1093/nar/gki005.

[2] Wyatt T. Clark and Predrag Radivojac. “Information-theoretic evaluation of predicted ontological annotations”. In: *Bioinformatics* 29.13 (2013), pp. i53–i61. ISSN: 13674803. DOI: 10.1093/bioinformatics/btt228.

[3] Naihui Zhou et al. “The CAFA challenge reports improved protein function prediction and new functional annotations for hundreds of genes through experimental screens”. In: *bioRxiv* (2019). DOI: 10.1101/653105.

[4] Predrag Radivojac et al. “A large-scale evaluation of computational protein function prediction”. In: *Nature Methods* 10.3 (2013), pp. 221–227. ISSN: 1548-7091. DOI: 10.1038/nmeth.2340. arXiv: 1510.05682.

[5] Aditya Grover and Jure Leskovec. *node2vec: Scalable Feature Learning for Networks.* 2016. arXiv: 1607.00653 [cs.SI].
